# Supplementary material for: Prevalence of hypertension among adolescents (10-19 years) in India: A systematic review and meta-analysis of cross-sectional studies
Source: PLoS One. 2020 Oct 6;15(10):e0239929. doi: 10.1371/journal.pone.0239929 (PMC7537899; doi:10.1371/journal.pone.0239929)
Supplement: S1 Table — (DOCX) [file pone.0239929.s002.docx]

**S1 Table: Search strategy**

| **Search engine** | **Keywords** |
| --- | --- |
| **PubMed** | #1- (Hypertension OR Essential hypertension OR Primary hypertension OR High blood pressure OR Elevated blood pressure OR Raised blood pressure OR Blood pressure)  #2- (Prevalence OR Epidemiology OR Risk)  #3- (Adolescents OR Children OR School going children)  #4- (India)  #5- Add 1 AND 2 AND 3 AND 4 |
| **Embase** | #1- ‘hypertension’/exp OR ‘blood pressure’/exp OR ‘high blood pressure’/exp OR ‘elevated blood pressure’  #2- ‘Prevalence’/exp OR ‘Epidemiology’/exp OR ‘Risk’  #3- ‘Adolescents’/exp OR ‘children’/exp OR ‘school going children’  #4- ‘India’  #5- #1 AND #2 AND #3 AND #4 |
| **Cochrane library** | #1- Hypertension OR High blood pressure OR Elevated blood pressure  #2-Prevalence OR Epidemiology OR Risk  #3- Adolescents OR Children  #4- India  #5- Add 1 AND 2 AND 3 AND 4 |
| **IndMed** | #1- “Hypertension OR  Primary hypertension OR Elevated blood pressure”  #2- “Prevalence OR Epidemiology”  #3- “Adolescents OR Children”  #4- “India”  #5- Add 1 AND 2 AND 3 AND 4 |
| **Google Scholar** | Search in Google Scholar was restricted up to 25 pages |
