# Supplementary material for: Prevalence of hypertension among adolescents (10-19 years) in India: A systematic review and meta-analysis of cross-sectional studies
Source: PLoS One. 2020 Oct 6;15(10):e0239929. doi: 10.1371/journal.pone.0239929 (PMC7537899; doi:10.1371/journal.pone.0239929)
Supplement: S2 Table — (DOCX) [file pone.0239929.s003.docx]

**S2 Table: Risk of bias assessment for all the selected studies for systematic review and meta-analysis**

| **S.No** | **Author & year of publication** | **Selection** | **Comparability** | **Outcome** | **Quality score** |
| --- | --- | --- | --- | --- | --- |
| 1. | Mohan et al. 2004 | 1 | 0 | 2 | 3 |
| 2. | Anjana 2005 | 2 | 0 | 2 | 4 |
| 3. | Saha 2007 | 3 | 0 | 3 | 6 |
| 4. | Savitha 2007 | 3 | 0 | 3 | 6 |
| 5. | Sharma 2009 | 3 | 0 | 3 | 6 |
| 6. | Goel 2010 | 4 | 2 | 3 | 9 |
| 7. | Khan 2010 | 3 | 0 | 3 | 6 |
| 8. | Buch 2011 | 2 | 0 | 3 | 5 |
| 9. | Durrani and Waseem 2011 | 4 | 0 | 3 | 7 |
| 10. | Mujumdar 2012 | 2 | 0 | 3 | 5 |
| 11. | Kumar 2012 | 4 | 2 | 3 | 9 |
| 12. | Yuvaraj 2014 | 3 | 2 | 3 | 8 |
| 13. | Lone 2014 | 4 | 0 | 3 | 7 |
| 14. | Anand 2014 | 3 | 0 | 3 | 6 |
| 15. | Faujdar 2014 | 4 | 0 | 3 | 7 |
| 16. | George 2014 | 3 | 2 | 3 | 8 |
| 17. | Garg 2015 | 3 | 0 | 3 | 6 |
| 18. | Mahajan and Negi 2015 | 4 | 0 | 3 | 7 |
| 19. | Kumar 2015 | 4 | 0 | 3 | 7 |
| 20. | Maiti and Bandyopadhyay 2016 | 3 | 1 | 3 | 7 |
| 21. | Reddy and Vamsheedar 2017 | 3 | 0 | 3 | 6 |
| 22. | Kumar 2017 | 5 | 0 | 3 | 8 |
| 23. | Singh et al. 2017 | 4 | 0 | 3 | 7 |
| 24. | Rai 2018 | 4 | 0 | 3 | 7 |
| 25. | Gupta et al. 2018 | 4 | 2 | 3 | 9 |
