# Supplementary material for: Prevalence of hypertension among adolescents (10-19 years) in India: A systematic review and meta-analysis of cross-sectional studies
Source: PLoS One. 2020 Oct 6;15(10):e0239929. doi: 10.1371/journal.pone.0239929 (PMC7537899; doi:10.1371/journal.pone.0239929)
Supplement: Attachment — Submitted filename: Response to reviewers.docx [file pone.0239929.s004.docx]

**Reply to the reviewers’ comment**

Prevalence of hypertension among adolescents (10-19 years) in India: A systematic review and meta-analysis of cross-sectional studies.

| **Reviewer Number** | **Original comments of the reviewer** | **Reply by the author(s)** | **Changes done on (manuscript with track changes) page number and line number** |
| --- | --- | --- | --- |
| 1 | I suggest to the authors to replace in the title the term “observational” by “cross-sectional” since in inclusion criteria, they considered only cross-sectional studies. | We thank the reviewer for this correction. This is corrected now | Page- 1, line-3 |
| 1 | Methods: The authors should update the stage of the review in PROSPERO. For instance, “Preliminary searches” are not “completed” | This is addressed now | Page-7, line-138 |
| 1 | Inclusion: It is not clear whether the authors only considered population/community-based studies (including school-based) studies. Only this kind of studies should be considered to have reliable epidemiological data | This is addressed now | Page-7, line-144. |
| 1 | Data extraction: Cochrane do not recommend scoring quality assessment | We agree, sir. However, there are instances where Cochrane reviewers have used Newcastle Ottawa Scale in systematic reviews of observational studies. The following is one example of a Cochrane review where NOS has been used.  **Reference**:  1. Lansbury LE, Rodrigo C, Leonardi-Bee J, Nguyen-Van-Tam J, Shen Lim W. Corticosteroids as Adjunctive Therapy in the Treatment of Influenza: An Updated Cochrane Systematic Review and Meta-analysis. Read Online: Critical Care Medicine \| Society of Critical Care Medicine [Internet]. 2020 [cited 2020 Apr 17];48: e98. | Page-8, line-168-175 |
| 1 | Statistical analysis: Consider reporting all prevalence estimates with 95% prediction intervals that better measure uncertainty | We believe confidence intervals are better suited for the present study, considering that the purpose of using prediction intervals is primarily for predicting future unobserved observation and for other reasons mentioned in the following citation:  **Reference:**  Chiolero A, Santschi V, Burnand B, Platt RW, Paradis G. Meta-analyses: with confidence or prediction intervals? Eur J Epidemiol [Internet]. 2012 [cited 2020 Apr 17]; 27:823–5. | Page-8 |
| 1 | Results: Characteristics of included studies. The sentence “All included studies were cross-sectional studies.” is not necessary since in the Inclusion criteria, the authors stated that they will consider only cross-sectional studies | This is addressed now | Page-9, line-206 |
| 1 | Table 1 should include diagnostic criteria for hypertension | This is addressed now | Page 11,12,13, Table 1. |
| 1 | Table 2: The signification of 0, 1, 2, 3, 4 for Selection, Comparability, and Outcome is not clear. This was not described any part in manuscript. | This is addressed now | Page-8, line-171-714 |
| 1 | Please report all prevalence with only one decimal | This is addressed now | Entire document |
| 1 | “a little increase”. I’m not sure it is adequate to write a little increase. I would suggest changing to “no substantial change”. | This is addressed now | Page-21, line-320 |
| 1 | “Analysis repeated after removing two studies that reported the hypertension prevalence of more than 15% and the prevalence of hypertension decreased from 7.64% (95% CI: 6.13% to 9.14) to 6.75% (95% CI: 5.55 to 7.96%) with more precise estimate.” It is not a correct methodological practice to exclude studies based on the outcome of outcome (here prevalence). Sensitivity analyses are carried out on methodological criteria, criteria for inclusion of participants and methodological quality (risk of bias). The authors may explore why these studies had higher prevalence when looking at participants characteristics, method (example sampling method), setting (urban vs rural for example)… Authors can conduct leave-one-out sensitivity analysis to assess the effect on the overall pooled estimate of removing each study. | This is addressed now | Page-21, line-321-328 |
| 1 | “We also reran the main analysis by removing two community-based study and one hospital-based study”. It is not clear why the authors removed both community-based and hospital-based studies. | This is addressed now | Page-21, line-321-328 |
| 1 | “The analysis was repeated after removing the study done by Maiti & Bandyopadhyay with sample size of less than 200 and the pooled estimate came out to be 7.57%, 95% CI: 6.04 to 9.09%.” The authors should give a strong rationale on the reason for choosing a threshold of 200. | This is addressed now | Page-21, line-321-328 |
| 1 | Table 5.  a. It is not whether the findings in this table are for univariable or multivariable meta-regression. Both models (uni- and multivariable) should be reported. b. I do not understand why some variance are reported with “(-)”. Variance is never negative. c. P value less than 0.05 should be reported with at least three decimals. d. Meta-regression should include methods to diagnose hypertension i.e. diagnostic criteria. e. For total no. of males and females, only the proportion of males or females should be considered since the no. can vary among studies, but the proportion is a comparable measure of the representativeness of sex distribution. f. How many studies were included in final multivariable model? g. How many studies were included in each univariable model? h. It is not clear why for the “Age group” variable, the authors reported only one coefficient since there would be n-1 coefficients according to the number age groups. | All points raised for this table are addressed now | Table 4, Page-22 |
| 1 | Discussion: “The possible reason for disparity in the estimate of hypertension prevalence might be due to the race and ethnicity of the developed and developing countries and the criteria used to classify hypertension”. What is the difference in term of ethnicity and race that explain this difference; i.e. what is the race/ethnicity in India and the race/ethnicity in the study by Moraes et al. In addition, also describe criteria to classify hypertension in both studies. And then discuss the mechanisms of these differences. | This is addressed now | Page-23, line-355-361 |
| 1 | The comparison with the study by Noubiap and colleagues should be more detailed. The authors suggested Black Africans children in Africa had lower prevalence compared to Indian Adolescents. Authors may report prevalence data on hypertension in adults and compare Indians and Black Africans to support this hypothesis (consider using global burden of diseases data) [https://jamanetwork.com/journals/jama/fullarticle/2596292]. Also add sentences on the mechanism of this difference: genetics? Dietary habits (with more details)? | This is addressed now | Page 23, line:365-372 |
| 2 | hard to understand what the authors are meaning | This is addressed now | Page-5, line- 105. |
| 2 | This should be the beginning of a new paragraph, starting with “Adolescents…” | This is addressed now | Page-5, line-109 |
| 2 | The authors explain the rationale of their study by indicating that most studies that were carried out in India were school based, and that their review would help solving such inconsistencies. I totally disagree, as the review is just a summary of existing literature; limitations of individual studies constitute limitations of the review… | This is addressed now | Page-6, line-113-116 |
| 2 | These sentences relate to study selection, not literature search strategy | This is addressed now | Page-7, line:140-141. |
| 2 | why only cross-sectional studies were included? Baseline or end-of-study data of cohort studies could present prevalence estimates, as well as control arms of RCTs… | We thank the reviewer for this comment. However, we believe that large cross-sectional studies would be the best design for this question, as even control arm of RCTs or cohort study population would not be representative. A cross-sectional study would be able to yield the best estimate for baseline risk.  **Reference**:  Kesmodel US. Cross-sectional studies – what are they good for? Acta Obstetricia et Gynecologica Scandinavica [Internet]. 2018 [cited 2020 Apr 7]; 97:388–93. | Page-7, line:142 |
| 2 | The title is about a study in adolescents, but here the authors say they have included children and ados up to 19yo | This is addressed now | Page-7, line:142-143 |
| 2 | please what do you mean by enough data was available? | This is addressed now | Page-7, line:144-145 |
| 2 | why papers written in Hindi were not considered? All these exclusions may have tended to increase the probability of publication bias | There are no databases for Hindi studies and all the Indian papers are written in English. The following is the reference for all papers from India being published in English. For these reasons we believe that this will not be a source of publication bias.  **Reference:**  Medical Journals of India [Internet]. [cited 2020 Apr 17]. Available from: http://medind.nic.in/ | Page-7, line:146 |
| 2 | why were studies within which parents had specific conditions rather than their kids excluded? | This is addressed now | Page-7, line:120-152 |
| 2 | I do not align with eliminating duplicates without verifying which is the more recent and complete version before ruling out | This is addressed now | Page-7, line:158-159 |
| 2 | I would have suggested to add age groups, setting (urban/rural), sex (male/female), and year of publication as other variables for subgroup analysis; social d; social development index/human development index or quintile of life are other parameters to consider, to know if poorer ados have more or less HTN than richer ones | This is addressed now | Page-9, line:186-187 |
| 2 | Figure1: there is a problem in this figure: records excluded should be linked with records after duplicates removed instead of records screened; in fact, it is after exclusion that the remaining records are screened What do you mean by non-relevant articles, n=92? Does it mean that your first round of exclusion was too sensitive? | This is addressed now | Figure 1 |
| 2 | Table1: Is putting the study area more informative than for eg the setting: urban vs rural? It is also important to know how HTN was defined in each of these studies; was the study multisite/national representative? | This is addressed now | Table1, Page-11 |
| 2 | majority is how many? | This is addressed now | Page-15, line:223 |
| 2 | how many studies were national representative, data were they collected prospectively or retrospectively? | This is addressed now | Page-15,  line-230-231 |
| 2 | Table 2 can be sent as supplementary table or the final score can be included in table 1 | This is addressed now | Table S2 (Supplement) |
| 2 | what guided the breakdown into 4 regions? This was not presented and clearly explained in methodology | This is addressed now | Page-9, line:186-187 |
| 2 | this is hard to understand: what was the region of reference, for the others to have significant different prevalence estimates? | This is addressed now | Page-19, Line-286-287 |
| 2 | the reason given to justify why publication bias was not assessed in the whole sample of studies is not clear to me. Should this bias be assessed only when there is low heterogeneity? | This is addressed now | Page-20, line:310-311 |
| 2 | Table5: how can males and females be included in the same model? This is to be the same variable, sex with two modalities male and female Study setting, region, method of sampling, method of HTN definition, etc could be explored in the model | This is addressed now | Table 4, page-22 |
| 2 | Discussion: no reference is given to support the statement on HTN and race; I am not sure the comparison between Noubiap and the authors is suitable, as Noubiap included children in their review | This is addressed now | Page-23, line-365-373 |
| 2 | Discussion: what is the pertinence in this statement? | This is addressed now | Page-24, line:377-378 |
| 2 | these lines are very insufficient as limitations of this study; please see comments above; in addition, discuss the generalizability of finding to the entire country, heterogeneity, etc | This is addressed now | Page-28, line:480-485 |
| 2 | Further, I think the discussion is insufficient: little was discussed about clinical implications of this study in terms of harmonizing the way of measuring BP and defining HBP among ados in the country; public health implications in terms of what strategies could be put in place to raise awareness on the issue and preventive measures/programs that could be introduced in school programs, etc | This is addressed now | Page-27-28, line-458-472 |
