# Supplementary material for: Prevalence of hypertension among adolescents (10-19 years) in India: A systematic review and meta-analysis of cross-sectional studies
Source: PLoS One. 2020 Oct 6;15(10):e0239929. doi: 10.1371/journal.pone.0239929 (PMC7537899; doi:10.1371/journal.pone.0239929)
Supplement: Attachment — Submitted filename: Response to Reviewers.docx [file pone.0239929.s005.docx]

**Response to reviewers**

Prevalence of hypertension among adolescents (10-19 years) in India: A systematic review and meta-analysis of cross-sectional studies.

| **Reviewer number** | **Original comments of the reviewer** | **Reply by the Author(s)** | **Changes done on page number and line number** |
| --- | --- | --- | --- |
| 1 | The authors stated that they will be included studies conducted among adolescents, 10 to 19 years. However, among studies included, some of them included participants < 10 years old: Anjana 2005 (6-14 years), Buch 2011 (6-18 years), Mujumdar 2012 (6-15 years), Yuvaraj 2014 (9-16 years), George 2014 (9-18 years), Kumar 2017 (5-14 years), and Rai 2018 (8-17 years). Definitively, this is not a study a meta-analysis of data from adolescents since the authors included data from children/infants. The authors should exclude these studies from the review or when possible extract data from adolescents only (> or = 10 years) (if not possible, then exclude). | Thank you for the comments. Yes, indeed as per inclusion criteria, we did include only the studies where it was possible to abstract data for ages 10 to 19 years. | Page 7, line 126-128. |
| 1 | The authors stated that they will include only population/community-based (including school-based) studies. This is an important criterion and I agree with this. However, the authors included a hospital-based study: Soundarssanane 2006 | Thank you so much. We have addressed this issue now. One hospital-based study is removed.  Accordingly, we updated our meta-analysis. | Page 10, updated table 1. After excluding study author Soundarssanane et al |
| 3 | The main title clearly identifies the report as a systematic review and meta-analysis; it also indicates the target population as well as the epidemiological measure (“prevalence”) that will be pooled. However, the running title does not contain the world “prevalence”. We suggest that “prevalence” should be added to the running title to fully match with the main title. | Thank you so much. This was an oversight from us. We have added the word ‘prevalence in title’ | Page 1, line 4. |
| 3 | Abstract- Background (line 43): The authors are right that there are concerns on the threat of elevated blood pressure in children and adolescents. It is also known that these concerns originate from existing evidence on the short-term and long-term ill effects of high blood pressure diagnosed in adolescence. This suggests that the authors could replace the expression “…considerable concerns on the threat…” by something like “… the well-known short-term and long-term ill effects…”. | Thank you for this suggestion. We have done correction accordingly in the abstract. | Page 3, line 39. |
| 3 | Methods: The processes of study search and selection were described. However, there is no mention that the study only included manuscripts written in English language (lines 48-50); it is a detail worth mentioning. Further, it is not stated that a meta-regression analysis was carried out, whereas the authors took great care to do it; this should be added to the abstract to accurately reflect the analyses performed during the research. | Thank you for this comment. This is addressed now | Page 3, line 46 & 51. |
| 3 | Results: The abstract contains the value of the pooled prevalence. It would have been interesting if it also contained the range of the prevalence across studies, so that readers could have an idea of the variability of such measure. Besides, the value of the heterogeneity of subgroup analyses was described as “acceptable” (line 59). It is understandable that a low level of heterogeneity can be deemed acceptable. However, it might be preferable to use the established thresholds of heterogeneity which will qualify an I-square = 18.3% as “small”. | Thank you for this suggestion. We have done correction accordingly in the abstract. | Page 3, line 54-57. |
| 3 | Conclusion: The authors proposed legitimate policy measures that should be put in place to control hypertension in adolescents. These recommendations could have been stronger if they were preceded by a comment describing the value of that pooled prevalence as “high”. | This is addressed now. We have now removed this recommendation from the manuscript. | Page 4, line 60-61. |
| 3 | #Key words (line 65): The abstract contains the key words that will facilitate future bibliographical searches. “Systematic review” and “prevalence” are other important key words that were omitted and ought to be added. | Thank you for this suggestion. We have done correction accordingly in the keywords. | Page 4, line 62. |
| 3 | (lines 88-90) The authors supported their point by raising awareness about the burden of cardiovascular diseases (CVD) in developing countries. The expression “…which is now being recognized as a major killer in developing countries” conveys the same information as the first part of the sentence and thus should be removed. Furthermore, the argument about high BP in adolescents of developing countries could have been strengthened by providing factual evidence such as the value of 5.5% that represents the pooled prevalence of elevated blood pressure in children and adolescents in Africa computed by Noubiap JJ et al. | Thank you for this comment. This is corrected now in the introduction. | Page 5, line 79 and line 86-87. |
| 3 | (lines 100-104) An attempt was made to provide evidence of the link between blood pressure in adolescence and hypertension in adulthood, which is commendable. We believe that more detailed information on this link should be presented to further support the rationale of this study. | Thank you so much for this comment. This is addressed now. | Page 5, line 94-97. |
| 3 | (lines 104-106) Childhood blood pressure (BP) was presented as the” best” indicator of adult BP. The quality of the reference used is not contested. However, the use of the word “best” is questionable, because it supposes that in this reference, measures of association were used to quantify the predictive value of childhood BP; it also suggests that a comparison was made with other potential predictive factors. In their report, Xiaoli Chen and Youfa Wang did a meta-regression analysis of correlation coefficients rather than measures of association. Further, they did not make a comparison with the predictive value of other potential predictive factors such as diet, family history, etc… Therefore, it would be more accurate to rather describe childhood BP as a “strong” indicator of adult BP. | Thank you for this comment. This is addressed now. | Page 5, line 98 |
| 3 | (lines 116-123) The importance of the current research is justified by arguing that there is a lack of studies reporting on the prevalence of hypertension among adolescents; it is also claimed that there exists no national survey investigating that issue. It is important to remind that the current work is not an original research but rather a review of existing studies; furthermore, it is not a national survey. Therefore, alternate arguments should be found to justify the relevance of the present study | Thank you so much for this comment. This is addressed now in the rationale part. | Page 6, line 105. |
| 3 | (lines 163-167) The process used to extract data was presented clearly. However, it is not possible to know if there were attempts made to contact the authors of publications that had missing information on key variables. This is critical given that 3,326 participants had missing information on sex (line 208), and 3 studies did not mention the number of BP readings (line 291). | Authors were not contacted. | - |
| 3 | (lines 168-174) The quality of each included study was assessed using an established scale. The 2 sentences spanning from line 168 to line 174 convey the same information and hence should be combined. | Thank you for this comment. This is addressed now. | Page 8, line 150-156. |
| 3 | (lines 182-183) There was an attempt to measure the heterogeneity between the included studies. The only metric used was the I-square. Although we do not contest that the I-square must be presented, using it solely is not convenient for several reasons: (1) the I-square is not a test statistic but rather a scale; (2) the I-square does not measure the heterogeneity itself but rather provides the percentage of variation across studies that is due to their heterogeneity rather than chance; (3) there is much uncertainty about the I-square when there are few studies such as is the case here (https://training.cochrane.org/handbook/current/chapter-10#section-10-10-2); (4) the established test statistic to confirm the presence of heterogeneity is the Cochrane's Q statistic. Therefore, in addition to the I-square, a Cochrane's Q statistic test of heterogeneity should also be performed, and the results presented and discussed. | Thank you so much for this suggestion. Cochrane's Q statistic test of heterogeneity is reported throughout the paper now. | Page 8, line 164 |
| 3 | (line 183) The result of the pooled analysis is referred to as the “mean” percent prevalence. It is agreed that the pooled analysis uses a sort of average across studies, but it is in no case a simple mean since there are specific weights that are applied. Therefore, the term “mean” should be replaced by “pooled”. | Thank you for the comment. This is addressed throughout the paper now. | Page 8, line 166 |
| 3 | (lines 198-192) A meta-regression analysis was carried out to identify the causes of heterogeneity. Two important information were not provided: (1) how the model fit was assessed; (2) how the researchers controlled for false-positive findings (type I error) when performing meta- regression with multiple covariates. This information should be provided to reinforce the robustness of the analyses seen throughout the paper. | Thank you so much for this valuable suggestion. This is addressed now.  1.) R^2^ value is reported for assessment of goodness of fit.  2.) Monte Carlo permutation test was conducted and the adjustment for type 1 error did not changed the result. | Page 9, line 177-179. |
| 3 | (line 212) Table 1 presents the characteristics of the studies included in the systematic review and meta-analysis. It appears that several of these studies included participants with an age that fell outside the target range of 10-19 years. This raises several questions about the following: (1) the accuracy of the study selection process, especially whether the inclusion criteria were respected; (2) the rationale of restricting the meta-analysis to the adolescent population given the rarity of studies treating about hypertension in the youth of India; this further raises concerns about the main title of the systematic review. | Thank you for the comments. Yes,  1.) As we have stated before we have included only those studies where it was possible to abstract data for ages 10 to 19 years. Thus, this suggestion is already incorporated in our review.  2.) We intended for results to be applicable to the adolescent age group since it represents a distinct demographic and a unique stage of development. For this reason, we included studies from which we could abstract data specific to this age group, and the title reflects this. | - |
| 3 | (line 206-207) It should be written: “The total number …was 28,355…” | Thank you for this comment. This is addressed now | Page 9, line 189 |
| 3 | (line 250) As stated above, the term “mean” should be replaced by “pooled”. | Thank you for this comment. This is addressed now | Page 15, line 216 |
| 3 | (line 252) As stated above, the I-square is not a heterogeneity test but rather a simple scale. The Cochrane's Q statistic test should be performed, and the results presented and discussed. | Thank you for this comment. As mentioned above we have addressed this throughout the manuscript. | Page 15 and page 17 – Table 2 and 3. |
| 3 | (Paragraph starting on line 352) There are valuable arguments presented to explain why the value of the pooled estimate is different from what other authors had found. It would have been easier for the readers to follow the trend of thoughts if there was first a formal comparison made between these values, before presenting the reasons for any difference observed. | Thank you for this comment. This is addressed now. | Page 19, line 282. |
| 3 | (line 392-393) Community-based studies were described as “superior” to school-based studies. The manuscript lacks the criteria on which this superiority was assessed as well as the references. | Thank you so much for this comment. This is addressed now. | Page 20, line 316-318. |
| 3 | (line 380-381) Specific references should be provided for the criteria of the European Hypertension society and the American Academy of Pediatrics, respectively. | Thank you for this comment. The references have been added now. | Page 20, line 307. |
| 3 | (line 411-428) The authors did well by highlighting that some of the differences observed might be due to the difference in blood pressure measurement methods used in various studies. However, a more thorough discussion on these methods is warranted because of the great influence that they might have on the accuracy of the diagnosis of hypertension. | Thank you so much for suggestion. This is addressed now. | Page 21, Line 347-367. |
